# The IMEx coronavirus interactome: an evolving map of *Coronaviridae*–host molecular interactions

**DOI:** 10.1093/database/baaa096

**Published:** 2020-11-18

**Authors:** L Perfetto, C Pastrello, N del-Toro, M Duesbury, M Iannuccelli, M Kotlyar, L Licata, B Meldal, K Panneerselvam, S Panni, N Rahimzadeh, S Ricard-Blum, L Salwinski, A Shrivastava, G Cesareni, M Pellegrini, S Orchard, I Jurisica, H Hermjakob, P Porras

**Affiliations:** European Molecular Biology Laboratory, Wellcome Genome Campus, European Bioinformatics Institute (EMBL-EBI), Hinxton, CB10 1SD, UK; Krembil Research Institute, Data Science Discovery Centre for Chronic Diseases, University Health Network, 5KD-407, 60 Leonard Avenue, Toronto, ON, M5T 0S8, Canada; European Molecular Biology Laboratory, Wellcome Genome Campus, European Bioinformatics Institute (EMBL-EBI), Hinxton, CB10 1SD, UK; European Molecular Biology Laboratory, Wellcome Genome Campus, European Bioinformatics Institute (EMBL-EBI), Hinxton, CB10 1SD, UK; UCLA-DOE Institute, UCLA, Los Angeles, CA 90095, USA; Department of Biology, University of Rome Tor Vergata, Via della Ricerca Scientifica, Rome, 00133, Italy; Krembil Research Institute, Data Science Discovery Centre for Chronic Diseases, University Health Network, 5KD-407, 60 Leonard Avenue, Toronto, ON, M5T 0S8, Canada; Department of Biology, University of Rome Tor Vergata, Via della Ricerca Scientifica, Rome, 00133, Italy; European Molecular Biology Laboratory, Wellcome Genome Campus, European Bioinformatics Institute (EMBL-EBI), Hinxton, CB10 1SD, UK; European Molecular Biology Laboratory, Wellcome Genome Campus, European Bioinformatics Institute (EMBL-EBI), Hinxton, CB10 1SD, UK; Department of Biology, Ecology and Earth Sciences, Università della Calabria, Rende, 87036, Italy; UCLA-DOE Institute, UCLA, Los Angeles, CA 90095, USA; Providence John Wayne Cancer Institute, Department of Translational Molecular, Santa Monica, CA 90404, USA; Univ Lyon, University Claude Bernard Lyon 1, INSA Lyon, CPE, Institute of Molecular and Supramolecular Chemistry and Biochemistry (ICBMS), UMR 5246, F-69622 Villeurbanne, 69622, France; UCLA-DOE Institute, UCLA, Los Angeles, CA 90095, USA; European Molecular Biology Laboratory, Wellcome Genome Campus, European Bioinformatics Institute (EMBL-EBI), Hinxton, CB10 1SD, UK; Department of Biology, University of Rome Tor Vergata, Via della Ricerca Scientifica, Rome, 00133, Italy; Department of Molecular, Cell and Developmental Biology, UCLA, Los Angeles, CA 90095, USA; European Molecular Biology Laboratory, Wellcome Genome Campus, European Bioinformatics Institute (EMBL-EBI), Hinxton, CB10 1SD, UK; Krembil Research Institute, Data Science Discovery Centre for Chronic Diseases, University Health Network, 5KD-407, 60 Leonard Avenue, Toronto, ON, M5T 0S8, Canada; Departments of Medical Biophysics and Computer Science, University of Toronto, Toronto, ON, M5T 0S8, Canada; European Molecular Biology Laboratory, Wellcome Genome Campus, European Bioinformatics Institute (EMBL-EBI), Hinxton, CB10 1SD, UK; European Molecular Biology Laboratory, Wellcome Genome Campus, European Bioinformatics Institute (EMBL-EBI), Hinxton, CB10 1SD, UK

## Abstract

The current coronavirus disease of 2019 (COVID-19) pandemic, caused by the severe acute respiratory syndrome coronavirus (SARS-CoV)-2, has spurred a wave of research of nearly unprecedented scale. Among the different strategies that are being used to understand the disease and develop effective treatments, the study of physical molecular interactions can provide fine-grained resolution of the mechanisms behind the virus biology and the human organism response. We present a curated dataset of physical molecular interactions focused on proteins from SARS-CoV-2, SARS-CoV-1 and other members of the *Coronaviridae* family that has been manually extracted by International Molecular Exchange (IMEx) Consortium curators. Currently, the dataset comprises over 4400 binarized interactions extracted from 151 publications. The dataset can be accessed in the standard formats recommended by the Proteomics Standards Initiative (HUPO-PSI) at the IntAct database website (https://www.ebi.ac.uk/intact) and will be continuously updated as research on COVID-19 progresses.

## Introduction

Severe acute respiratory syndrome, or SARS, emerged as a life-threatening viral disease of unknown origin in late 2002 in the Guangdong Province of southern China caused by the severe acute respiratory syndrome coronavirus (SARS-CoV) (1). The SARS-CoV-2 is a related virus responsible for the current outbreak of coronavirus disease of 2019 (COVID-19) (2). As of September 2020, over 26.5 million people globally have been shown to be infected with the virus, with more than 873 000 deaths directly attributed to its effects (worldometer, https://www.worldometers.info/coronavirus/). To distinguish the strains that caused the SARS outbreak of 2002 from other SARS-related, nonhuman-hosted SARS-CoV strains, we refer to the former as SARS-CoV-1.

The COVID-19 pandemic has resulted in massive scientific efforts attempting to fight the disease and understand the biology of the virus. This has resulted in enormous challenges for the research scientist when attempting to find and select information relevant to specific areas of viral biology and pathology. In order to aid the scientific community and expedite drug and vaccine development, multiple data curation efforts have been undertaken to perform a critical assessment of the literature and represent different aspects of the virus and the disease in a structured and computationally accessible manner. One recent example of such efforts is the COVID-19 Disease Map (3), a community effort to capture the intricate aspects of SARS-CoV-2 biology as reusable and interoperable pathway maps, so they can be used in systems biology and modeling pipelines.

Identification of virus–host interactions and the analysis of the topological structure of a relevant molecular interaction network are the necessary steps to understand the cellular mechanisms involved in a biological process such as viral infection of a cell. A detailed map of the interactions between human and pathogen proteins will aid in a more complete awareness of the mechanisms of infection and subsequent viral replication, assembly and release and may help to identify novel drug targets, or assist in a rapid and more accurate repurposing of existing drugs for treating or preventing infection. Further to this, such networks can be used to study changes in the transcriptome or proteome of a virally infected cell when compared to normal cells. Co-regulated genes or proteins that also co-cluster in this network may indicate that these entities are involved in the same biological process or are members of the same functional complex.

For such a network to be of value to the researcher, a certain amount of metadata needs to be provided, which enables the assessment of network quality and data types. These data need to be supplied in a standardized and computer-accessible format, which allows for scoring, filtering and selection. The International Molecular Exchange (IMEx) Consortium (4) has been providing such data for over 15 years, supplying experimental details using controlled vocabulary terms, captured using a detailed curation model. Important aspects of an interaction experiment are described, including host organism, interaction detection and participant identification methodologies as well as details of the constructs such as binding domains and the effects of site-directed mutations. Current membership of the IMEx Consortium includes the IntAct (5), MINT (6), DIP (7), UniProt (8), MatrixDB (9) and IID (10) data resources, who collaborate to provide the users with a single, consistent viral–host dataset to work with. When a novel virus emerges, as was the case with SARS-CoV-2 in 2019, the study of closely related species, such as SARS-CoV-1 and other coronaviruses, may help with this process, giving the scientific community time to produce species-relevant data. For this reason, the network includes data on all coronaviruses available in the scientific literature. While primarily consisting of protein–protein interaction data, the network also contains interactions with lipids, glycosaminoglycans and RNAs, again curated to IMEx standards. The data are open under a CC-BY 4.0 license and downloadable from the IntAct website and FTP in PSI-MI standard tab-delimited and XML-based formats. A brief description can be found at http://www.ebi.ac.uk/intact/resources/datasets, and a collection of interactive network representations of the dataset at the time of writing is available at http://www.ndexbio.org/#/networkset/4c2268a1-a0f0-11ea-aaef-0ac135e8bacf. The dataset will be expanded and updated with every IntAct release.

## Methods

### Data sources

All statistics and details shown on this manuscript are derived from the IntAct database release of 2020-07-11. Complex Portal annotations used in Supplementary Table 4 were also obtained from the same data release.

Specific analyses include the following datasets: SARS-CoV-2—all human targets of SARS-CoV-2 viral proteins; SARS-CoV-1—all human targets of SARS-CoV-1 viral proteins; Gordon_LT—all human targets of SARS-CoV-2 viral proteins derived by Gordon *et al.* plus selected SARS-CoV-2 low-throughput (LT) studies; Li_LT—all human targets of SARS-CoV-2 viral proteins derived by Li *et al.* plus selected SARS-CoV-2 LT studies and Stukalov_LT—all human targets of SARS-CoV-2 viral proteins derived by Stukalov *et al.* plus selected SARS-CoV-2 LT studies. The selected LT studies added to Gordon, Li and Stukalov studies represent interactions that were detected in studies with less than 50 interactions and not detected in the high-throughput (HT) ones. Such interactions involve SARS-CoV-2 Spike (interacting with seven human proteins, such as ACE2, BSG, DEFA5, FURIN, CCNB1, CLEC4M and CD209), nsp3 (interacting with human PARP10) and nsp8 (interacting with human HLA-A). Formatted subsets are available in Supplementary Table 5. We also included in Supplementary Table 1D a reduced version of the dataset where only *Coronaviridae* protein interactions are included and interactions with human proteins are highlighted.

### Analysis software and packages

**Figure 1.**
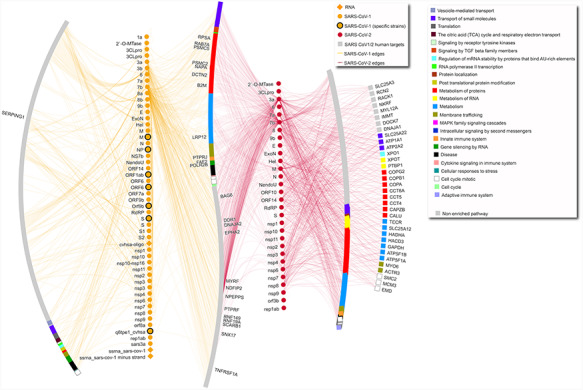
SARS-CoV-1–human and SARS-CoV-2–human network. The network includes only interactions between SARS-CoV-1/2 proteins and human targets. Human targets are annotated with level-one Reactome ontology, as shown in legend. Only targets with degree equal or higher than 5 are labeled. Diamond SARS-CoV-1 nodes represent RNA molecules. SARS-CoV-1 specific strains include strains PC4-145, Frankfurt 1, Urbani, TJF, HB and GZ02. Gray nodes are annotated with pathways that are not enriched in this network. In the network, the leftmost arc shows human proteins targeted only by SARS-CoV-1 proteins, the vertical line made of yellow nodes indicates SARS-CoV-1 proteins, the S-shaped arc shows human proteins targeted both by SARS-CoV-1 and SARS-CoV-2 proteins, the vertical line made of red nodes indicates SARS-CoV-2 proteins, and the two right arcs show proteins targeted only by SARS-CoV-2, with the rightmost arc including only proteins with degree equal or higher than 5.

Analyses were done using R 4.0.0 (11) and the R data.table package (12). Venn diagrams were created using ggVennDiagram 0.3 (13). All other plots were created using ggplot2 3.3.0 (13) and wesanderson (14) packages. The network in Figure 1 was created using NAViGaTOR 3.0.13 (15); the data used to create the network are provided in Supplementary Table 5F. The rest of the interactive networks were created using Cytoscape 3.8.0 (16), and all networks were uploaded to NDEx using Cytoscape.

### Tissue enrichment analysis

Analysis was performed using TissueEnrich (17) 1.8.0 R package on human protein targets using Protein Atlas (18) expression data. Background was the entire proteome as listed in the UniProtKB website (https://www.uniprot.org/proteomes/UP000005640). Only hits with a fold change above zero are shown. Log10 *P*-value was zero for all tissues. Full data are available in Supplementary Table 3.

### Pathway enrichment analysis

Pathway enrichment analysis was performed using pathDIP 4 (19) API in R, with literature curated set. Only pathways with false discovery rate (FDR) < 0.01 (Benjamini Hochberg (BH)-method) were considered. As the majority of enriched pathways (75% for SARS-CoV-1, 80% for SARS-CoV-2, 72% for Gordon_LT, 76% for Li_LT and 55% for Stukalov_LT) were from Reactome database (20), we further organized them using level-one Reactome ontology to create the figure. All enriched pathways are listed in Supplementary Table 2, and pathways present in multiple sets are highlighted in the ‘overlap’ tab. For network annotation, each protein was linked to the pathway it is part of with the lowest FDR (BH-method) and annotated with level-one Reactome ontology according to such pathway. When a protein was annotated with two different level-one terms (this happened only for 58 targets in common between SARS-CoV-1 and SARS-CoV-2), only the SARS-CoV-2 level-one term was used.

## Results and discussion

As of August 2020, the dataset contains 3735 unique interacting molecule pairs, represented in 4479 binarized interactions extracted from 151 publications, 8 of which are preprints from bioRxiv. The entire ‘coronavirus dataset’ can be downloaded from the FTP site in PSI-MI standard XML-based formats: PSI-MI XML 2.5 and 3.0 (format documentation available at https://psicquic.github.io/PSIMIXML.html). Each file corresponds to one publication in the coronavirus dataset. In addition to the XML format, the data can also be browsed on the IntAct webpage (www.ebi.ac.uk/intact/query/annot:“dataset:coronavirus”), where it is available for download in additional formats, such as the tab-delimited PSI-MI-TAB 2.7 (format documentation available at https://psicquic.github.io/MITAB27Format.html). Viral specific interactomes can also be retrieved using MIQL syntax. For example, to filter for human-SARS-CoV-2 interactions, the string ‘taxid:2697049 AND taxid:9606’ can be used in the search box.

### NDEx archive

A snapshot of the coronavirus dataset hosted in IntAct at the time of writing is also available as a collection of interactive network representations in the NDEx archive. Here, we submitted five networks, each highlighting a key aspect of the dataset:

- Full dataset in collapsed view, with unique molecule pairs linked by edges representing aggregated evidence: https://doi.org/10.18119/N9MP4S
- Full dataset and full evidence details represented as multiple edges: https://doi.org/10.18119/N9RC8F
- Full dataset, highlighting evidence where mutant forms of proteins involved are available: https://doi.org/10.18119/N9W590
- Full dataset, highlighting evidence where experimental information about binding regions of proteins involved are available: http://doi.org/10.18119/N90W3W
- Subset of SARS-CoV-1 and SARS-CoV-2 to human targets interactions with pathway annotation (Figure 1): https://doi.org/10.18119/N9831P.

Details about the visual styles used to depict evidence, species and further information are provided in the NDEx network description and in Supplementary ‘methods’ section.

### Other access options

Using the same search strings as on the IntAct website, the data can also be accessed via the PSICQUIC (21) API, the IMEx Consortium webpage (www.imexconsortium.org) and the VirusMentha (22) browser at https://virusmentha.uniroma2.it/.

### As with all data hosted by IntAct, the dataset is available under the CC BY 4.0 license

The complete dataset includes all the interactions that have been found in publications that fulfill the criteria reported in the next paragraph. The data refers mostly to protein–protein interactions (3568 interactions), plus some interactions involving different types of RNA (84 interactions) or small molecules (57 interactions). While data on 81 organisms are included (Supplementary Table 1A), most interactions refer to SARS-CoV-2–human and SARS-CoV-1–human interactions (2019 and 870 unique interactions, respectively). The dataset of only human–*Coronaviridae* interactions is highlighted in Supplementary Table 1D. Figure 1 shows SARS-CoV-2– and SARS-CoV-1–human interactions, separating human targets that are specific for one or the other coronavirus or in common between the two, also highlighting affected pathways.

IMEx Consortium curators have collated interaction evidence from scientific articles and preprints using the following selection criteria:

- The publication contains interactions involving proteins from any virus member of the *Coronaviridae* family (NCBI taxon ID 11118). This includes not only SARS-CoV-1 and SARS-CoV-2, but also Middle East Respiratory Syndrome (MERS)-CoV and members of the family that infect other mammals.
- The publication contains interactions of human proteins with established relevance for SARS-CoV-2 life cycle (e.g. ACE2 interactions have been included in the dataset).
- Every interaction described in these publications is curated and included in the dataset, even if their relevance to COVID-19 might seem limited. This results in the inclusion of data from apparently irrelevant species (e.g. yeast) that is of interest from the phylogenetic and evolutionary point of view.
- Preprints are considered and, if deemed appropriate, curated when containing SARS-CoV-2 data, an exception to IMEx practice of representing only peer-reviewed research. This reflects the interest these data have engendered during the pandemic. These datasets are clearly marked as prepublication and will be re-curated, if necessary, when published.

**Figure 2.**
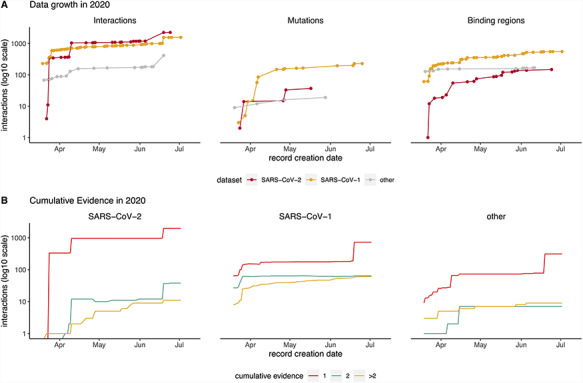
Timeline showing data captured in IMEx resources since COVID-19 outbreak (March 2020). (A) Cumulative interactions, mutation features and binding regions annotated for SARS-CoV-2 (red), SARS-CoV1 (orange) and other *Coronaviridae* family members proteins (gray). Interactions include spoke-expanded binary relationships. Dots represent the date when the interaction was curated. (B) Amount of cumulative experimental evidence associated with unique binary pairs, captured over time, in each of the three datasets: SARS-CoV-1, SARS-CoV-2 and other *Coronaviridae* family members. Interactions include spoke-expanded binary relationships.

The IMEx curation model captures the details of specific constructs used for the detection of interactions. This allows for the representation of different construct-associated features, such as specific mutations affecting interaction outcome (23) or sequence regions that are associated with binding. Most of these in-depth studies are centered around the Spike–ACE2 interaction for SARS-CoV-2 and SARS-CoV-1. The only other SARS-CoV-2 mutation reported so far is Nsp5/3C-like proteinase p.Cys145Ala catalytically inactive mutant, which exhibits an interaction profile different from the canonical form in an affinity purification and mass spectrometry (AP-MS) study (24). There are a few other mutations reported for SARS-CoV-1 and in other members of the *Coronaviridae* family, but it is clear that specific variation effects on coronavirus-related interactions is an area requiring extensive exploration. As shown in Figure 2A, fragment constructs have been used in more studies than mutations, although some of these might have been designed for convenience reasons (e.g. constructs that are easier to express in heterologous systems). Interactive network representations highlighting mutations and binding regions can be found in https://doi.org/10.18119/N9W590 and in https://doi.org/10.18119/N90W3W.

Regarding interaction data generation approaches, the experimental setup used to detect an interaction is summarized by three key fields in the IMEx curation model: the method used to determine that an interaction is happening (‘interaction detection method’), the method used to determine which molecules are involved (‘participant detection method’) and the biological environment in which the interaction occurs (‘host organism’). Each of these is described by an appropriate controlled vocabulary term, making the data readily searchable. In the coronavirus dataset, the overwhelming majority of data were generated by AP-MS approaches performed in A549, HEK293(T) or HeLa cells (Supplementary Table 1B). This type of data produces sets of potential interacting partners (preys) associated with a bait of interest but does not directly identify binary interacting partners. Also, the data needs to be automatically expanded into binaries for its representation in tabular or network graph form. This accounts for a large proportion of binary interactions from spoke-expanded n-ary relationships found in the dataset: 3267 out of 4477 interactions (73%) are expanded n-ary relationships, while only 41% of the binary interactions found in the full IntAct database are expanded n-ary relationships.

**Figure 3.**
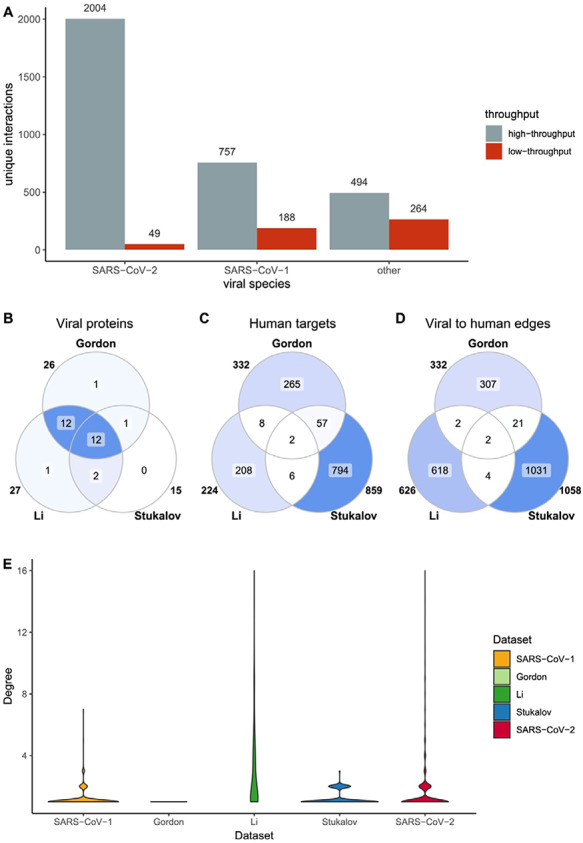
SARS-CoV-2 HT datasets comparison. SARS-CoV-2 datasets include Gordon *et al*., Li *et al*. and Stukalov *et al*. (A) Distribution of HT/LT-derived interactions in the Coronavirus dataset. The number on top of each bar indicates the number of publications per category. HT publications are defined as those hosting more than 50 unique interacting pairs. (B-D) Overlap of viral proteins (B), human targets (C) and viral to human edges (D) across datasets. Numbers outside the circles indicate the size of the sets. (E) Distribution of number of interactions between viral proteins and human targets. In the y-scale, 1 indicates that one human protein interacts only with one viral protein, while 16 indicates that one human protein interacts with 16 viral proteins.

Most content has been curated after declaration of the COVID-19 pandemic on 11 March 2020 (Figure 2). The data growth timeline shows four jumps in interaction numbers, three for SARS-CoV-2 and one for SARS-CoV-1, due to curation of HT studies not available for other *Coronaviridae* (Figure 3A). This is also reflected in the pattern of data growth regarding detailed information about mutations and binding regions (Figure 2A). The plots also illustrate that at this point there are more studies dealing with detailed interaction data for SARS-CoV-1, a situation that will likely change as more SARS-CoV-2 studies are performed.

Interaction data are derived from both, small-scale studies focused on one or just a few interactions (LT) and large-scale screenings able to detect hundreds or thousands of interactions in a single experiment (HT). The extreme urgency in the study of SARS-CoV-2 has resulted in a strong dominance of HT data for this species in comparison with the other members of *Coronaviridae* reported in the dataset (Figure 3A). Additionally, SARS-CoV-2 small-scale studies are mainly focused around the ACE2–Spike interaction due to its relevance for virion recognition and infection. For the remaining interactions, most SARS-CoV-2 data comes from three studies: Stukalov *et al*. (25), where A549 cell lines were transduced with viral proteins followed by AP-MS analysis, and Gordon *et al*. (24) and Li *et al*. (26), both focused on AP-MS detection of interacting candidates in HEK293(T) cells transfected with SARS-CoV-2 proteins. The studies are distinct, showing small overlap (Figure 3B–D) with each other and no overlap at all with small-scale studies. They also show different degree distribution patterns (Figure 3E). Gordon *et al*. shows an unexpected pattern of fully isolated components, likely due to stringent target selection.

Lack of overlap between different HT interaction datasets is a long-recognized phenomenon, since different experimental approaches are better suited to detect interactions featuring specific physicochemical characteristics and protein abundances, among other parameters (27–29). Even in this case, where the approach used is broadly similar, they likely reflect methodological differences. AP-MS datasets are very sensitive to protein abundances and affinities, as well as to the selection and orientation of tags and expression systems. Additionally, strong differences can arise during the selection of *bona fide* interactors, where multiple strategies can be used in order to clean up spurious detections. The lack of common interactions between the different studies focused on SARS-CoV-2 suggests that more of these systematic experiments are needed to increase reliability of biological conclusions extracted from this type of data.

**Figure 4.**
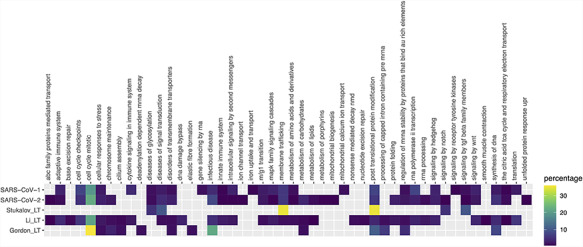
Pathway enrichment analysis of SARS-CoV-1, SARS-CoV-2 and Gordon_LT (Gordon plus low throughput), Li_LT (Li plus low throughput) and Stukalov_LT (Stukalov plus low throughput) datasets. Enrichment was performed using pathDIP (a database integrating 24 different pathway databases). Only human proteins were considered. The majority of enriched pathways were from Reactome database, so a mapping of each Reactome pathway to the parent pathway ontology was performed, and the heat map shows the percentage of pathways in each parent pathway over the total of pathways.

Exploring the biological context of SARS-CoV-2 interactors suggests that the three HT studies are complementary. Pathway enrichment (Figure 4 and Supplementary Table 2) finds commonalities on expected pathways related with cell cycle, response to stress and infectious disease, and molecules synthesis and processing. Key pathways such as innate immune response or cytokine signaling are only found in the dataset of Li *et al.*, along with several intracellular signaling and metabolic routes. Only SARS-CoV-1 shows enrichment in lung tissue, but none of the sets reach the 5× enrichment threshold suggested by Jain and Tuteja (17) (Supplementary Figure 1, Supplementary Table 3). Finally, SARS-CoV-2 interactors from the three studies are found as components of exosome, deacetylases, ATPase transmembrane complexes and other mitochondrial complexes annotated in the Complex Portal (Supplementary Table 4) (30). We see an abundance of complexes involved in endocytosis in the study of Gordon *et al.*, protein production, Ca^2+^-dependent cell signaling and cell cycle control are mostly represented in the dataset provided by Li *et al.* while proteins involved in transcriptional regulation and transport have been found in the interactome provided by Stukalov and collaborators.

Development of the IMEx coronavirus dataset complements other curation efforts that have been initiated in the light of the pandemic. Interaction resources BioGRID (31) and VirHostNet (the latter specialized in virus–host interaction data) (32) have produced shallow curation interaction datasets taken from COVID-19-related literature. The IMEx Coronavirus dataset has been used by the DisGeNET database (33) for contextual annotation with related diseases (https://www.disgenet.org/downloads#section9). Also, members of the IMEx Consortium are involved in the COVID-19 Disease Map initiative (https://covid.pages.uni.lu/), where interaction information from the dataset is guiding COVID-19-related pathway curation. As an example, the list of PMIDs from IMEx Coronavirus dataset has been used to screen papers containing causal interactions to build COVID-19 causal network perturbed during SARS-COV2 infection by the SIGNOR 2.0 resource (34) (https://signor.uniroma2.it/covid/) and to select the Gordon *et al.* human interactors to integrate in the SIGNOR 2.0 network.

We are also involved in a parallel effort of curating protein complexes from SARS-CoV-1, SARS-CoV-2, MERS-CoV as well as their human target complexes in the Complex Portal (https://www.ebi.ac.uk/complexportal), linking to available experimental interaction evidence in IntAct when possible. This initiative is especially relevant because, as previously stated, coronaviruses increase the number of functional proteins produced by the viral genome by posttranslational cleavage of long polypeptide transcripts. The functionality and/or stability of these proteins are further increased through the formation of protein complexes, all of which have been catalogued into the Complex Portal. To date, 14 complexes have been identified in each strain, formed by viral–viral protein interactions, including homomeric assemblies such as the dimeric SARS-CoV-2 main protease complex (CPX-5685). Other reference entities such as the SARS-CoV-2 Spike–human ACE2 receptor complex (CPX-5683) have also been created to enable their identification in large omics datasets. All complexes have been annotated with Gene Ontology terms, describing their role in the virus lifecycle, which again will assist in the analysis of large omics-derived datasets.

## Conclusions

Accurate and detailed representation of biological insight into public databases is a fundamental source of data for scientific discovery, even more so in a situation of accelerated research work such as the current pandemic. Our curation of molecular interactions related to *Coronaviridae* enables a systematic perspective of this data and greatly increases its interoperability. From our overview analysis of the data available so far, we can highlight how the SARS-CoV-2 data seem to be both biologically relevant, and thus informative for research on the disease, and strongly preliminary, so full consideration for its inherent incompleteness should be given when using it. The IMEx Consortium is expanding the resource as new data become available, striving to provide the most accurate possible picture of the *Coronaviridae* interactome.

## Supplementary Material

baaa096_Supp
