# Supplementary material for: The IMEx coronavirus interactome: an evolving map of Coronaviridae–host molecular interactions
Source: Database (Oxford). 2020 Nov 18;2020:baaa096. doi: 10.1093/database/baaa096 (PMC7673336; doi:10.1093/database/baaa096)
Supplement: baaa096_Supp [file baaa096_supp.zip › supplementary_methods.docx]

Supplementary Methods

**NDEx networks detailed legend**

**IMEx / IntAct Coronavirus Dataset, collapsed edges**

Full Coronavirus dataset as extracted from IntAct on July 2020.

*Edges:* Each edge is a composite of multiple physical interaction evidence.

Edge colour represents MIscore, a normalized characterization score ranging from 0 to 1 and weighting all the evidence available behind an interacting pair (1). Darker colour corresponds to a higher MIscore value (better characterized interactions).

Edge width represents the number of individual experiments in which the interaction was found.

*Nodes*: Nodes represent interacting molecules, which can be proteins, nucleic acids, small molecules, molecular complexes (multimolecular machineries) or molecular sets (groups of UniProt accessions representing proteins of identical sequence but generated from different genes).

Node colour represents different species origin, with SARS-CoV-2 painted in red, SARS-CoV-1 in orange and human in blue. The colour of the rest of a total of 81 species can be accessed by clicking on a node of interest in the NDEx network.

Node shape represents different molecule types:

- Circle: proteins and peptides
- Diamond: nucleic acids (DNA and different RNAs)
- Triangle: small molecules
- Octagon: molecular complexes
- Rounded square: molecule sets

Finally, SARS-CoV-2 and SARS-CoV-1 proteins are represented as larger nodes for ease of identification.

**IMEx/IntAct Coronavirus Dataset, full detail**

Full Coronavirus dataset as extracted from IntAct on July 2020.

*Edges:* Each edge represents a separate piece of evidence (a single experiment) for an interaction.

Edge colour representing different interaction types according to the PSI-MI Controlled Vocabulary (PSI-MI CV codes are provided between brackets):

- Green: ‘association’ [MI:0914]
- Blue: ‘physical association’ [MI:0915]
- Red: ‘direct interaction’ [MI:0407] and children, including enzymatic reactions
- Grey: ‘colocalization’ [MI:0403]

Edge width represents MIscore (see above), with thicker edges mapping to higher MIscore values.

Dashed line edges represent computationally-expanded binaries that have been extracted from interactions where more than two participating molecules have been reported. These are expanded into binaries by IntAct using the spoke-expansion algorithm(2).

*Nodes*: same representation schema provided for the collapsed edges network described above.

**IMEx/IntAct Coronavirus Dataset, mutations details**

Full Coronavirus dataset as extracted from IntAct on July 2020, highlighting mutation information. Nodes and edges where information about proteins with mutations affecting the interaction has been recorded are highlighted over the rest of the network.

*Edges:* Each edge represents a separate piece of evidence (a single experiment) for an interaction. Experiments where a mutant form for either or both of the proteins involved has been reported are highlighted in strong pink colour, while the rest of the edges have been greyed and dimmed.

The rest of edge visual characteristics are depicted as described in the 'full detail' network.

*Nodes*: Nodes use the same representation schema described for previous networks, except for mutation-affected nodes which are highlighted with a strong pink rim while unaffected nodes are dimmed.

**IMEx/IntAct Coronavirus Dataset, binding regions details**

Full Coronavirus dataset as extracted from IntAct on July 2020, highlighting binding regions information. Nodes and edges where information about proteins with interaction binding regions has been recorded are highlighted over the rest of the network.

*Edges*: Each edge represents a separate piece of evidence (a single experiment) for an interaction. Experiments where a binding region for either or both of the proteins involved has been reported are highlighted in green colour, while the rest of the edges have been greyed and dimmed.

The rest of edge visual characteristics are depicted as described in the ‘full detail’ network.

Nodes: Nodes for which binding information is available are highlighted with a green rim. The rest of the nodes are dimmed. In everything else, nodes use the same representation schema described for previous networks.

**IMEx SARS-CoV-1/2 - human Subset**

Human-SARS-CoV-1 and SARS-CoV-2 interactions extracted from IntAct on July 2020. Only interactions between SARS-CoV-1 or SARS-CoV-2 molecules and human proteins have been used for this network. Diamond nodes represent RNA molecules. Node annotations include level-one Reactome ontology (“category”). SARS-CoV-1 or SARS-CoV-2 molecules have been coloured following previous networks colour scheme. The same colours have been used for edges departing from either set of nodes, in order to clearly highlight interactions belonging to each viral strain. Human nodes have been coloured according to the level one Reactome ontology they are annotated with. The colour codes for pathway annotations correspond to the ones in Figure 1.

**References**

1. Villaveces,J.M., Jiménez,R.C., Porras,P., Del-Toro,N., Duesbury,M., Dumousseau,M., Orchard,S., Choi,H., Ping,P., Zong,N.C., *et al.* (2015) Merging and scoring molecular interactions utilising existing community standards: tools, use-cases and a case study. *Database (Oxford)*, **2015**.

2. Bader,G.D. and Hogue,C.W.V. (2002) Analyzing yeast protein-protein interaction data obtained from different sources. *Nat. Biotechnol.*, **20**, 991–997.
