# Supplementary figures and images for: The IMEx coronavirus interactome: an evolving map of Coronaviridae–host molecular interactions
Source: Database (Oxford). 2020 Nov 18;2020:baaa096. doi: 10.1093/database/baaa096 (PMC7673336; doi:10.1093/database/baaa096)

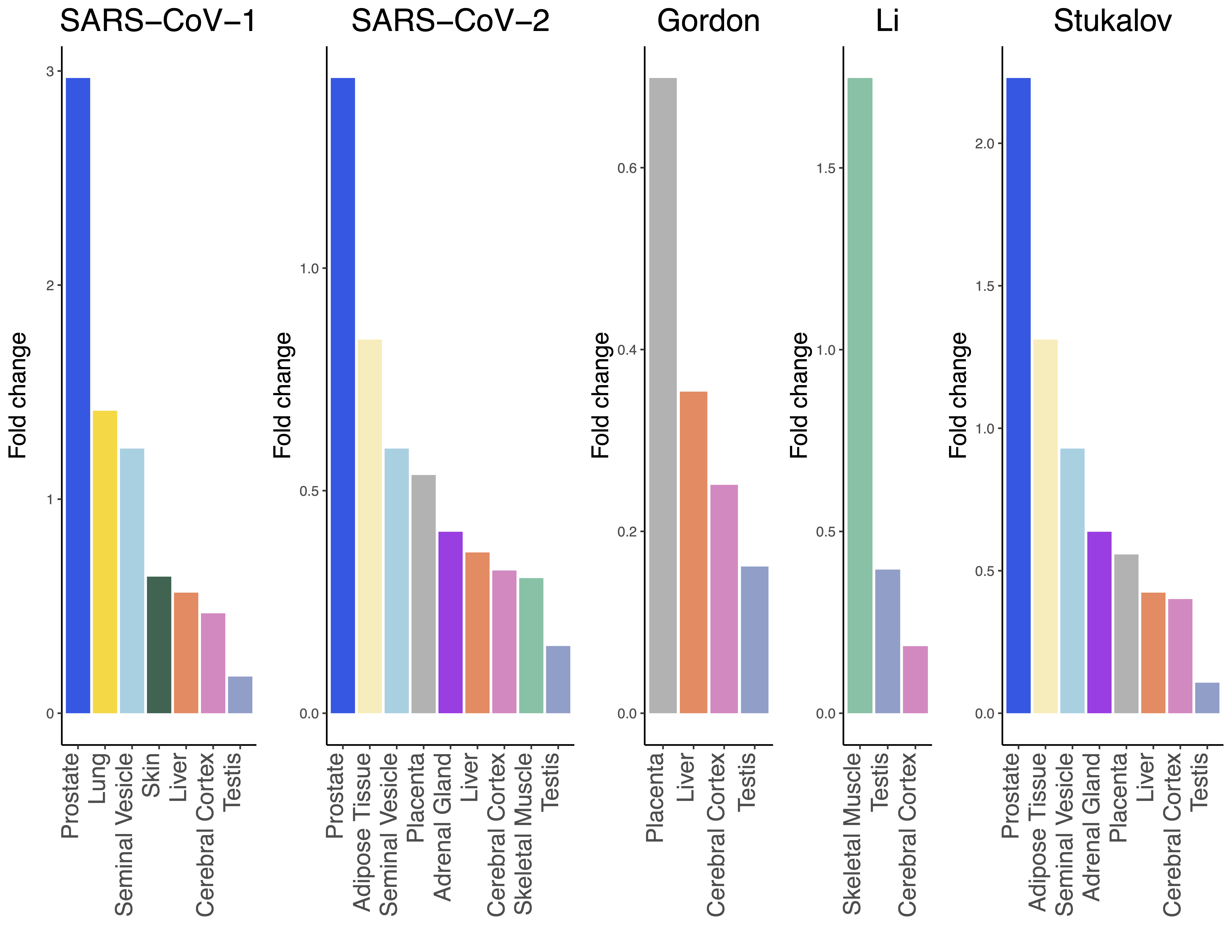

Supplement: baaa096_Supp [file baaa096_supp.zip › Supplementary_figure_1.jpg]
